# Under-recognized, under-referred: a multidisciplinary evaluation of fragility fracture management in the emergency setting

**DOI:** 10.1007/s11657-026-01684-y

**Published:** 2026-06-18

**Authors:** Francesco Bertoldo, Fabio De Iaco, Carlo Trevisan, Andrea Marcellusi, Ombretta Viapiana, Giovannella Baggio, Giorgio De Conti, Ferdinando Silveri, Antonella Cocorocchio, Sandro Giannini

**Affiliations:** 1 Emergency Medicine Unit, Department of Medicine, University of Verona, Verona, Italy; 2 Emergency Department, Ospedale Maria Vittoria, Turin, Italy; 3 Orthopedics and Traumatology Complex Unit, ASST Bergamo Est, Bergamo, Italy; 4 Department of Pharmaceutical Science (DISFARM), University of Milan, Milan, Italy; 5 Complex Operative Unit of Rheumatology, Department of Medicine, University of Verona, Verona, Italy; 6 Italian Research Center for Gender Health and Medicine, University of Padua, Padua, Italy; 7 Complex Operative Unit Radiology, Department of Integrated Diagnostic Services, University Hospital of Padua, Padua, Italy; 8 Rheumatology Clinic, Marche Polytechnic University, Ancona, Ancona, Italy; 9 Scientific Committee of FEDIOS (Federazione Italiana Osteoporosi e Malattie dello Scheletro), San Benedetto del Tronto, Italy; 10 Emergency Medicine and Surgery Department, San Giovanni Addolorata Hospital, Rome, Italy; 11 Medical Clinic 1, Department of Medicine, University of Padua, Padua, Italy

**Keywords:** Fragility fracture, Osteoporosis, Emergency medicine, Bone health, Risk assessment

## Abstract

***Summary*:**

Fragility fractures are frequently under-recognized in emergency departments. A survey of 34 professionals revealed inconsistent assessment of key risk factors. Relevant gaps emerged between awareness and routine practice across professional roles. These findings highlight opportunities to improve recognition, documentation, and secondary prevention in ED settings.

**Purpose:**

Fragility fractures are often under-recognized in the emergency setting, where the absence of standardized procedures leads to missed opportunities for diagnosis and secondary prevention. This project aimed to explore current emergency department (ED) practices regarding the identification and management of fragility fractures.

**Methods:**

A qualitative, cross-sectional survey was conducted among 34 healthcare professionals (10 emergency physicians, 11 radiologists, and 13 emergency nurses) from various Italian regions. The questionnaire, developed by a multidisciplinary expert panel, investigated propensities and self-reported practices related to fracture assessment, risk factor identification, and diagnosis of fragility fracture. All responses were grouped by role and represented graphically. Findings were then discussed during an expert meeting with the same panel.

**Results:**

Survey data revealed a generally high level of self-reported awareness of fragility risk factors among ED professionals but inconsistent implementation of the necessary diagnostic workup in daily practice. Important indicators, such as prior fractures, history of falls, family history of hip fracture, and use of risky medications, were often under-assessed. The term “fragility fracture” at discharge from the ED was rarely used, and role-based discrepancies emerged in risk assessment practices.

**Conclusion:**

This survey highlights relevant gaps between awareness and clinical practice in the identification of fragility fractures in the emergency setting. Improving consistency in risk assessment and documentation may represent a key step toward optimizing secondary prevention and standardizing fragility fracture management in EDs.

**Supplementary Information:**

The online version contains supplementary material available at 10.1007/s11657-026-01684-y.

## Introduction

Fragility fractures, defined as fractures resulting from low-energy trauma such as a fall from standing height or less, are a hallmark of underlying osteoporosis and represent a substantial public health challenge globally. They predominantly affect older adults and are associated with increased morbidity, long-term disability, mortality, and significant healthcare costs [1]. Despite their clinical significance, fragility fractures are often under-recognized and inadequately managed in the emergency department (ED), the most common setting in which fracture diagnosis is made [2]. This gap in recognition contributes directly to suboptimal secondary prevention, leaving patients vulnerable to subsequent fractures that further compromise quality of life and increase healthcare burden [3, 4].

Beyond their clinical consequences, fragility fractures impose a substantial economic and societal burden. In Europe, the direct costs of osteoporotic fractures were estimated to exceed €37 billion annually, with projections indicating a sharp rise due to population aging [5]. In Italy, a cost-of-illness analysis estimated the annual economic burden of osteoporosis at €2.2 billion in 2017, with approximately 80% of costs attributable to hospitalizations, 16% to drug treatments, and 3% to outpatient care [6]. Importantly, this estimate likely under-represents the true societal impact, since it does not fully capture indirect costs, productivity losses, and the economic burden borne by informal caregivers. Evidence suggests that these components can be substantial, amplifying the long-term financial implications of fragility fractures for both patients’ families and healthcare systems [5, 7–9].

Emergency departments serve as crucial points of contact for patients with fragility fractures, offering a unique but under-utilized opportunity for early identification, risk stratification, and prompt initiation of multidisciplinary management strategies [2]. However, the fast-paced and acute-driven environment of emergency care can impede comprehensive evaluation and timely referral to specialist services such as fracture liaison services (FLS) and osteoporosis clinics [4].

Recent literature underscores the vital role of multidisciplinary collaboration, encompassing emergency medicine, orthopedics, geriatrics, endocrinology, rehabilitation, nursing, and primary care in improving patient outcomes through integrated fracture management pathways [1, 5, 6]. Evidence supports the implementation of practical, validated scoring systems and screening tools that can be feasibly applied in the emergency setting to facilitate early identification of high-risk individuals [4]. These tools aim to bridge the current care gap by enabling targeted referrals and personalized interventions aimed at reducing the risk of refracture [3, 10].

This paper synthesizes current evidence on the multidisciplinary evaluation of fragility fracture management in the emergency setting and examines barriers to effective care coordination and risk recognition in ED practice. By highlighting these challenges, the study aims to contribute to improved identification of fragility fractures, more consistent referral pathways, and ultimately more effective secondary prevention and long-term patient outcomes.

## Methods

A cross-sectional survey (Supplementary materials 1) was conducted to investigate current practices, awareness, and perceived barriers in the identification and management of fragility fractures in emergency departments. A total of 34 healthcare professionals participated in the survey, including 10 emergency physicians, 11 radiologists, and 13 emergency nurses. The participants were drawn from 10 emergency departments distributed across northern, central, and southern Italy, including first- and second-level departments. The participating centers were based in Naples, Carpi, Milan, Venezia, Foggia, Rome, Ragusa, Reggio Emilia, and Turin, ensuring a broad geographic representation of emergency care settings across Italy. Collectively, these facilities handled approximately 450,000 emergency department visits in 2024. The survey consisted of 16 structured questions designed to explore how different professional figures assess and manage a case of fracture in the emergency setting: They are multiple-choice and open-ended questions covering areas such as patient triage, fracture risk factor assessment (e.g., history of falls, prior fractures, and use of risk-associated medications), interprofessional communication, and discharge practices. The instrument also explored whether existing workflows supported the identification of patients with potential bone fragility. Responses were collected through structured interviews conducted by trained facilitators. Following the survey, a focused expert board, composed of bone health specialists and emergency medicine physicians, reviewed and discussed the results. The purpose of the meeting was to critically review the survey findings, compare them with clinical experience, and explore practical solutions to address identified gaps. Key themes discussed included the discrepancy between reported and actual practices, the under-utilized role of nurses in history taking, and the limited involvement of radiologists in fragility risk assessment. Participants expressed strong support for the need to improve awareness, consistency, and standardization in the assessment of fragility fracture risk within emergency settings.

The outcome of this process is the identification of key domains and risk factors relevant to fragility fracture recognition, in line with evidence and variables included in validated algorithms such as FRAX and DeFRA [11–14].

## Results

Developed by a multidisciplinary panel composed of an emergency physician, an endocrinologist, specialists in internal medicine, a radiologist, an orthopedic surgeon, a rheumatologist, an emergency nurse, a health economist, and a gender medicine expert, the questionnaire covered a range of critical topics: recognition of clinical risk factors (such as previous fractures, family history, fear of falling, and balance impairment), evaluation of pharmacological risks (e.g., corticosteroids or hormonal therapies), documentation practices, availability of internal care pathways, and role-specific behaviors and perceptions. It also investigated potential barriers to standardized assessment and explored the perceived utility of a brief screening tool to support early identification and referral. Responses were analyzed by professional role to identify variability in attitudes, workflows, and clinical priorities and served as the foundation for a multidisciplinary expert discussion that followed.

**Fig. 1:**
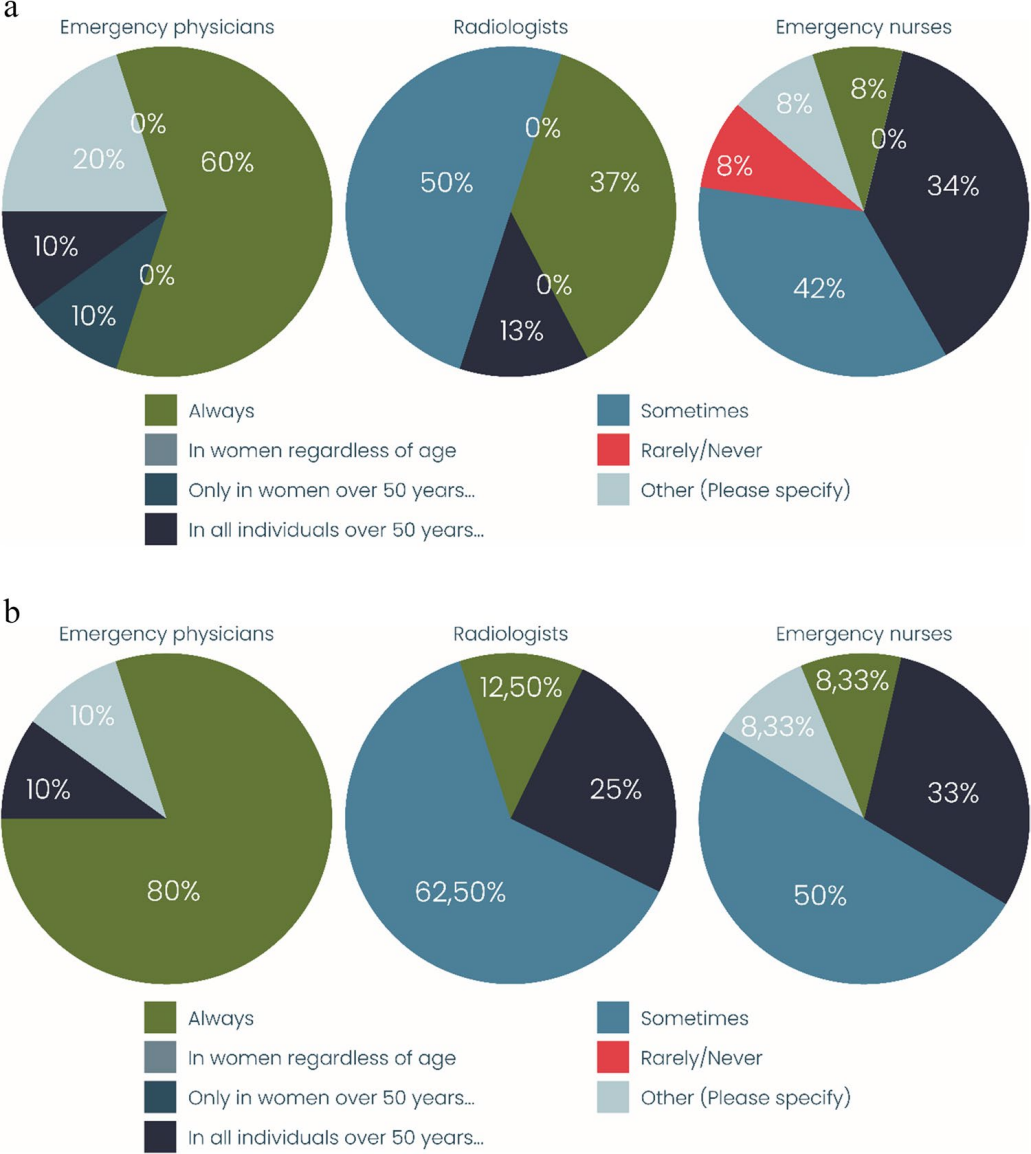
**a** Answers to question 1.2: Is a history of recent or frequent falls, and the mechanism of the fall, investigated? **b** Answers to question 1.3: Is there an investigation into a history of frequent or recent fractures and how they occurred?

Figure 1a shows how professionals investigate a history of recent or frequent falls and their dynamics. Emergency physicians reported the highest rate, with 60% stating they always assess this, compared to 37% of radiologists and 8% of nurses. Nurses showed greater variability, with a significant share responding “sometimes” or “in all individuals over 50 years of age.” These results highlight a stronger focus among physicians, while in other roles the assessment appears less systematic, potentially limiting early identification of fragility risk.

In line with the question on falls, responses to the investigation of frequent or recent fractures (Fig. 1b) showed a similarly uneven approach. Among emergency physicians**,** 80% reported “always” investigating prior fractures**,** reflecting a strong awareness of fracture history as a risk factor. In contrast, only 12% of radiologists and 9% of nurses reported the same level of attention. The majority in these two groups selected “sometimes” or “in all individuals over 50,” suggesting that the investigation is often limited to specific populations and not part of standard practice across the board.

**Fig. 2:**
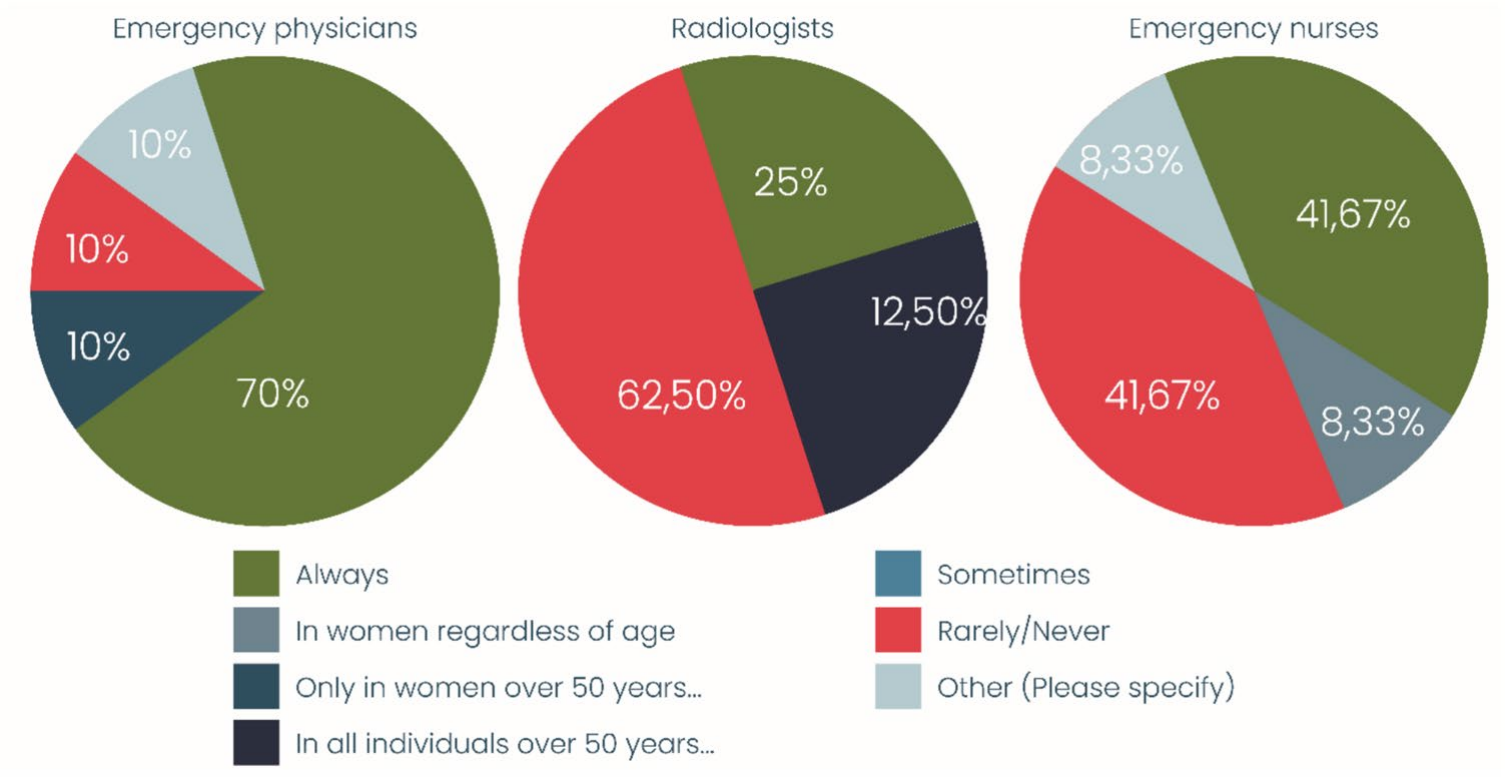
Answers to question 1.13: Is the patient’s medication history investigated as a risk factor for fractures (with particular attention to steroids, anticonvulsants, or adjuvant anti-hormonal therapies for cancer)?

The investigation of medication history as a risk factor for fractures, particularly regarding corticosteroids, anticonvulsants, or adjuvant hormone therapy, revealed clear differences across professional roles (Fig. 2). Among emergency physicians, a strong majority reported that they “always” assess this risk factor, with only 10% indicating “rarely” or only in women over 50 years. In contrast, radiologists showed a markedly different pattern: 22% reported doing so “always,” while 60% indicated they rarely or never investigate this information. Nurses showed intermediate behavior, with 40% responding “always” and 40% selecting “rarely or never.” These results highlight the critical role emergency physicians and nurses may play in identifying pharmacological contributors to bone fragility, while radiologists remain largely uninvolved in this aspect of clinical evaluation.

**Fig. 3:**
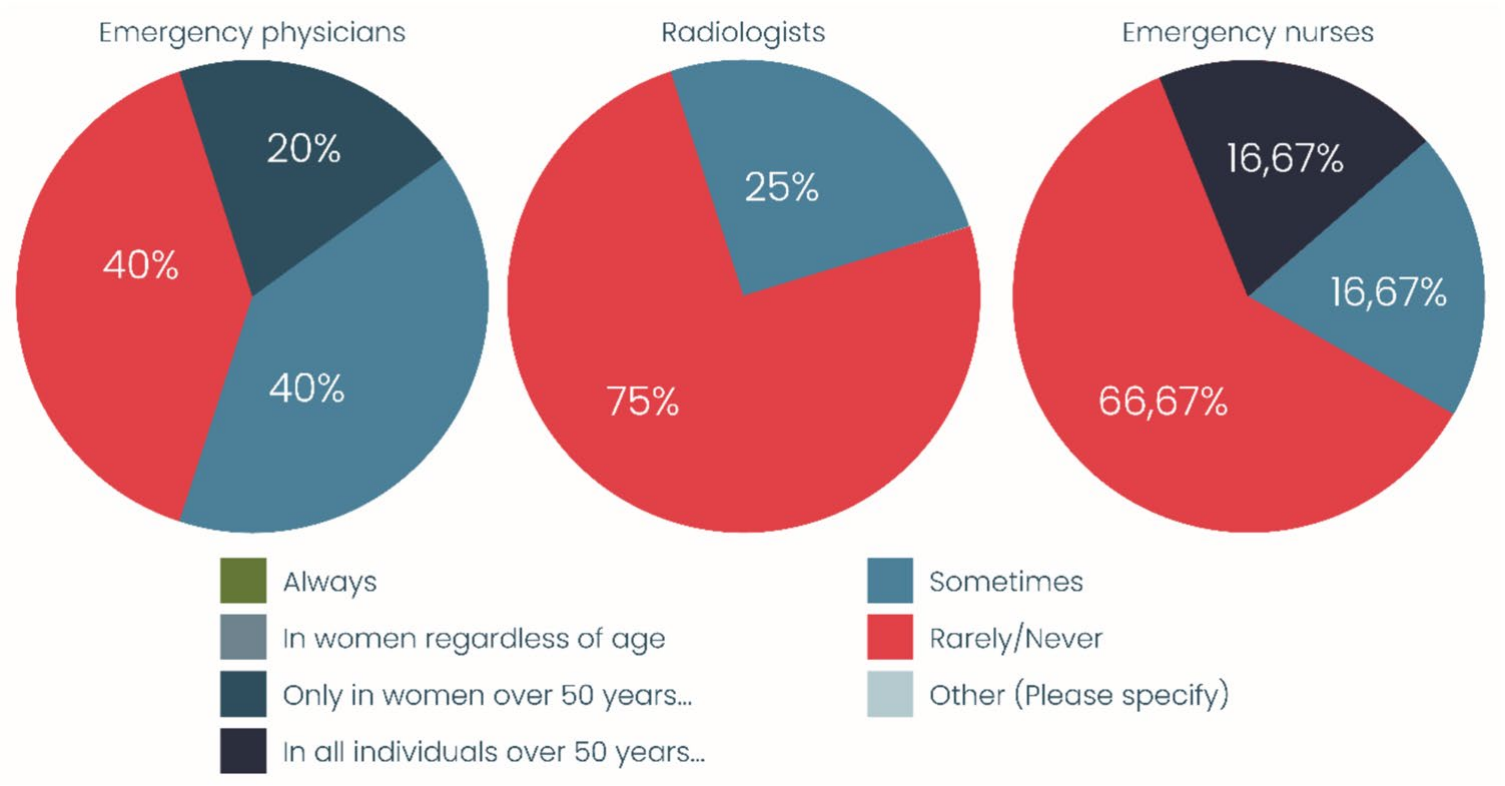
Answer to question 1.5: Do you investigate whether there are cases of hip or other fractures in the family, such as in first-degree relatives?

Assessment of family history of fracture is another important element in identifying patients at risk of osteoporosis. The investigation of family history of fractures, such as hip or other fracture sites in first-degree relatives, was reported inconsistently across all professional roles (Fig. 3). Among emergency physicians, the most frequent responses were “sometimes” (40%) and “rarely/never” (40%), indicating that this aspect is not routinely assessed even by frontline clinicians. Radiologists and nurses showed a similar trend, with the majority also reporting low or occasional investigation of familial risk. These findings show a general low attention to family history in the three professional categories even if at different levels.

**Fig. 4:**
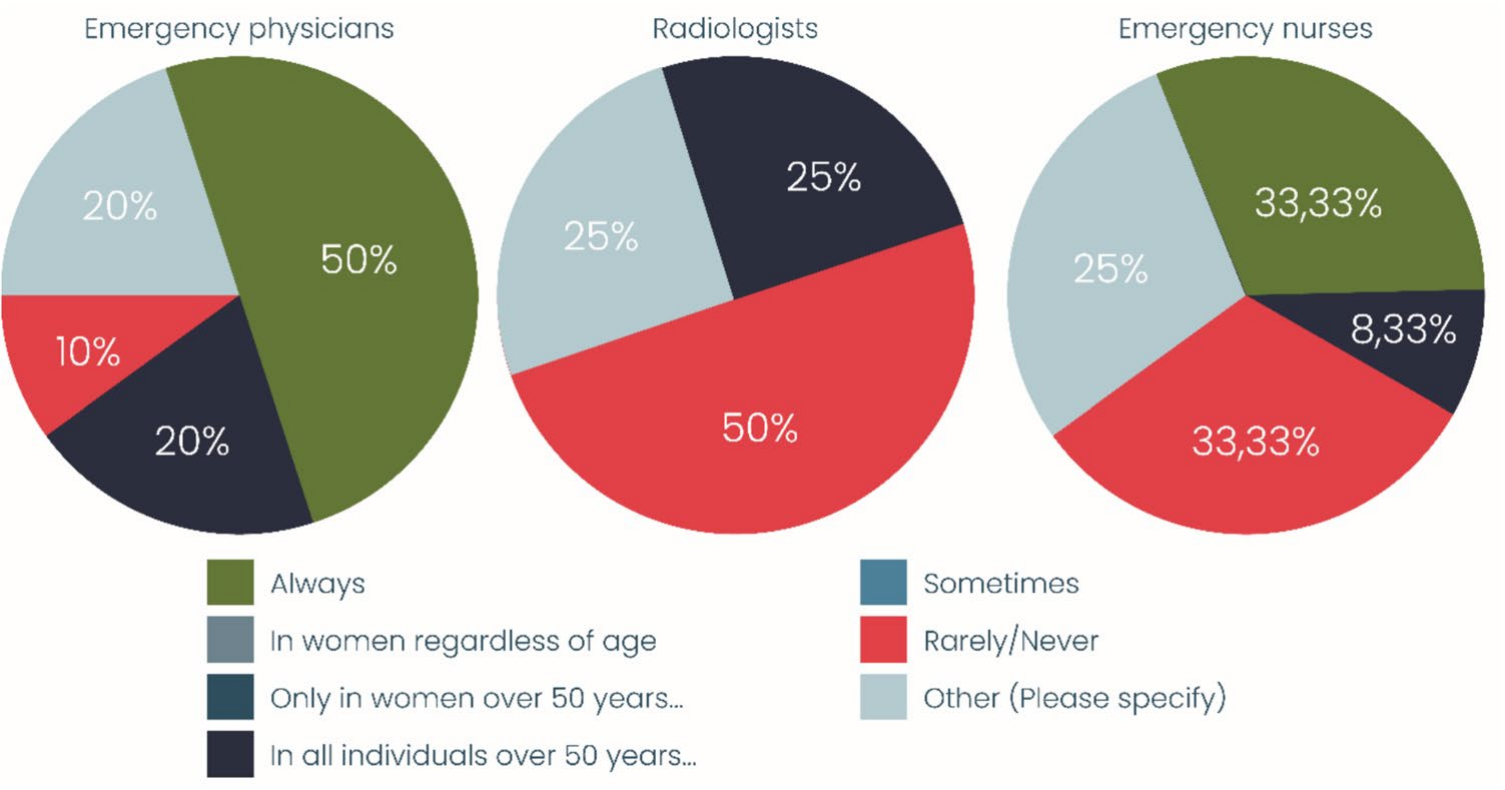
Answer to question 1.16: Do you investigate whether the patient has ever been afraid of falling or has noticed difficulty maintaining balance?

Fear of falling and self-reported balance issues are well-known predictors of future fractures and functional decline, particularly in older adults. However, these indicators are not routinely explored in the emergency setting. The assessment of fear of falling or difficulty maintaining balance is inconsistently performed across professional roles (Fig. 4). Among emergency physicians, only 50% reported “always” investigating this factor, while the remaining answers were split between “sometimes” and responses limited to specific subgroups. Radiologists showed minimal engagement, with most responses indicating “rarely/never” or “only in individuals over 50.” In contrast, emergency nurses showed a polarized pattern of responses, with only 30% reporting they “always” assess this aspect, while another 30% indicated they “rarely or never” investigate it. These results suggest that, although balance and fall-related fears are clinically relevant for fracture risk, they are variably integrated into emergency assessment routines.

The explicit use of the term “fragility fracture” in clinical documentation is critical for the start of the most appropriate pathway. In fact, when asked only to the emergency physicians how frequently they indicate a “fragility fracture” diagnosis at discharge (question n 1.21 Supplementary Materials), 80% responded “sometimes,” while only a small minority selected “never” (10%) or “other” (10%). Notably, none of the respondents reported indicating it “always.”

Across most items, the survey revealed inconsistencies between professional roles and between declared practices and what typically occurs in real-world settings. These findings were critically discussed during the expert panel meeting, where organizational barriers, role-specific limitations, and lack of standardized tools were identified as major contributors to the therapeutic gap.

In response, the panel discussed the practical implications of the identified gaps and emphasized the importance of early recognition of fragility fractures in postmenopausal women and men over 50 presenting with specific major fracture types (vertebra, pelvis, femur, humerus, and radius/ulna). The discussion highlighted the need for timely identification of patients at high risk of bone fragility and for structured referral to dedicated care pathways, ensuring appropriate follow-up, specialist evaluation, and secondary prevention.

Overall, the expert discussion underscored the clinical relevance of well-established fragility fracture risk factors already recognized in the literature [15–20] and commonly included in validated risk assessment frameworks, highlighting their inconsistent evaluation in routine emergency practice.

## Discussion

This study offers a qualitative overview of how fragility fractures are identified and managed in emergency settings, based on the perspectives of emergency physicians, radiologists, and nurses from 10 Italian hospitals. While general awareness of osteoporosis appears high across roles, the survey highlights a critical gap between knowledge and clinical practice, particularly in the systematic investigation of risk factors such as prior falls, previous fractures, medication use, and family history. One of the most relevant findings is the lack of uniformity in assessing fall and fracture history. Even though emergency physicians tend to investigate these aspects more consistently than nurses and radiologists, responses across all roles reveal that these assessments are neither standardized nor embedded into clinical workflows. Nurses, often the first point of contact in the ED, frequently limit their history taking to specific populations, and radiologists rarely explore fragility-related risk factors, despite their potential role in identifying low-trauma fractures. Similarly, the investigation of pharmacological risk and family history remains limited, especially outside emergency medicine. This inconsistency undermines early recognition and delays entry into appropriate care pathways.

Another key insight is the near-absence of the term “fragility fracture” in discharge documentation. This omission limits both the patient’s awareness of their underlying condition and the ability of downstream providers, such as primary care physicians and fracture liaison services (FLS), to recognize the fracture as a sentinel event requiring secondary prevention. In the absence of structured follow-up systems or automatic alerts, this gap in communication compromises timely initiation of osteoporosis management and contributes to the widening of the therapeutic gap in fragility fracture care. These findings are consistent with previous literature showing that many fragility fractures remain unrecognized in acute care, with the first fracture often failing to prompt appropriate follow-up [21]. Unlike other acute conditions such as stroke or myocardial infarction, where triage pathways and structured referral protocols are well established, fragility fractures continue to follow the same undifferentiated route as traumatic fractures. This leads to delays in appropriate treatment and prevents timely prevention strategies, despite the known consequences of recurrent fragility fractures on patient outcomes and healthcare systems. The expert panel’s discussion emphasized the need to address the identified gaps through a more structured and shared approach to fragility fracture recognition in the emergency setting. Strengthening early clinical suspicion and improving integration of fragility-related considerations into routine fracture assessment may support more appropriate referral pathways and enhance continuity of care.

This perspective aligns with the successful use of structured clinical approaches in other acute domains and supports the long-term goal of improving consistency in fragility fracture identification within emergency departments. Beyond the clinical and organizational implications, our findings also highlight an important economic dimension. Fragility fractures represent a substantial burden for healthcare systems, largely driven by hospitalizations and subsequent rehabilitation [5, 7]. In Italy, the annual economic impact of osteoporosis has been estimated at approximately €2.2 billion, of which nearly 80% is attributable to hospital care, while only a small fraction relates to outpatient visits or pharmacological treatment [6]. Failure to identify and label fragility fractures in the emergency setting, as observed in our survey, is therefore not only a missed clinical opportunity but also a source of inefficient resource use. Recurrent fractures increase the likelihood of repeated admissions and long-term dependency, thereby amplifying both direct healthcare costs and indirect costs associated with loss of independence and caregiver burden.

This study presents several limitations that should be acknowledged. First, the methodology was qualitative in nature, based on self-reported survey responses rather than objective measurement of clinical practice or outcomes. As such, the results provide insight into declared attitudes and behaviors but do not allow for verification of their consistency with actual clinical performance. Second, a notable discrepancy emerged between reported practices and what is known from the literature about real-world behavior in emergency settings, particularly regarding the roles of radiologists and nurses in assessing fragility risk factors, highlighting the risk of overreporting desirable actions. Third, the survey was not anonymous, which may have further contributed to response bias, especially among professionals less directly involved in patient history taking. Fourth, we did not collect diagnostic data or case-level fracture information, as the purpose of the project was to explore attitudes and organizational practices rather than to evaluate clinical prevalence or appropriateness of diagnosis. Lastly, the emergency department itself represents a highly specific and pressured environment, where time constraints, role fragmentation, and acute care priorities may limit the feasibility of conducting comprehensive risk assessments, regardless of clinicians’ awareness or intentions.

## Conclusion

This study highlights a persistent gap in the recognition and documentation of fragility fractures in emergency departments, despite increasing awareness of their clinical and organizational relevance. The findings underscore the lack of standardized approaches to distinguish fragility-related fractures from traumatic ones at the point of first contact, resulting in missed opportunities for timely referral and secondary prevention. The multidisciplinary discussion highlighted the need for a more structured and shared approach to the assessment of fragility fractures in the emergency setting. Improving consistency in fracture evaluation and early clinical suspicion may facilitate more appropriate care pathways and contribute to reducing the burden of secondary fractures and associated healthcare costs [6].

## Supplementary Information

Below is the link to the electronic supplementary material.

Supplementary file1 (DOCX 16.3 KB)

## Data Availability

The data used in this study are the property of Abiogen and are stored in a proprietary database maintained by the company.

## References

[1] Sáez-López P, Aldecoa Álvarez-Santullano C, Arboiro-Pinel R et al (2025) Recommendations for the prevention of fragility fractures: a consensus from international experts and Ibero-American scientific societies. Arch Osteoporos 20:76. 10.1007/s11657-025-01551-2 40504279 10.1007/s11657-025-01551-2 PMC12162770

[2] Jackson LE, Skains RM, Mudano A et al (2024) An emergency department-based system intervention to improve osteoporosis screening for older adults at high-risk of fracture. JBMR Plus 8:ziae038. 10.1093/jbmrpl/ziae038 38681999 10.1093/jbmrpl/ziae038 PMC11055962

[3] Javaid MK (2021) Efficacy and efficiency of fracture liaison services to reduce the risk of recurrent osteoporotic fractures. Aging Clin Exp Res 33:2061–2067. 10.1007/s40520-021-01844-9 34047929 10.1007/s40520-021-01844-9 PMC8302543

[4] Scholten DJ, Bray JK, Wang KY et al (2020) Implementation of a fracture liaison service and its effects on osteoporosis treatment adherence and secondary fracture at a tertiary care academic health system. Arch Osteoporos 15:80. 10.1007/s11657-020-00736-1 32468516 10.1007/s11657-020-00736-1

[5] Hernlund E, Svedbom A, Ivergård M et al (2013) Osteoporosis in the European Union: medical management, epidemiology and economic burden: a report prepared in collaboration with the International Osteoporosis Foundation (IOF) and the European Federation of Pharmaceutical Industry Associations (EFPIA). Arch Osteoporos 8:136. 10.1007/s11657-013-0136-1 24113837 10.1007/s11657-013-0136-1 PMC3880487

[6] Marcellusi A, Rotundo MA, Nardone C et al (2020) Osteoporosis: economic burden of disease in Italy. Clin Drug Investig 40:449–458. 10.1007/s40261-020-00904-8 32248346 10.1007/s40261-020-00904-8

[7] REFReSH study group, Leal J, Gray AM et al (2016) Impact of hip fracture on hospital care costs: a population-based study. Osteoporos Int 27:549–558. 10.1007/s00198-015-3277-9 10.1007/s00198-015-3277-9 PMC4740562 26286626

[8] Cheung W, Shen W, Dai D et al (2018) Evaluation of a multidisciplinary rehabilitation programme for elderly patients with hip fracture: a prospective cohort study. J Rehabil Med 50:285–291. 10.2340/16501977-2310 29260234 10.2340/16501977-2310

[9] Locke S, Doonan J, Jones B (2024) Advancements in the management of fragility fractures in orthopaedic patients. Cureus. 10.7759/cureus.74065 39712828 10.7759/cureus.74065 PMC11661880

[10] Walters S, Khan T, Ong T, Sahota O (2017) Fracture liaison services: improving outcomes for patients with osteoporosis. CIA 12:117–127. 10.2147/CIA.S85551 10.2147/CIA.S85551 PMC5237590 28138228

[11] Schini M, Johansson H, Harvey NC et al (2023) An overview of the use of the fracture risk assessment tool (FRAX) in osteoporosis. J Endocrinol Invest 47:501–511. 10.1007/s40618-023-02219-9 37874461 10.1007/s40618-023-02219-9 PMC10904566

[12] Adami G, Biffi A, Porcu G et al (2023) A systematic review on the performance of fracture risk assessment tools: FRAX, DeFRA, FRA-HS. J Endocrinol Invest 46:2287–2297. 10.1007/s40618-023-02082-8 37031450 10.1007/s40618-023-02082-8 PMC10558377

[13] Bonaccorsi G, Messina C, Cervellati C et al (2018) Fracture risk assessment in postmenopausal women with diabetes: comparison between DeFRA and FRAX tools. Gynecol Endocrinol 34:404–408. 10.1080/09513590.2017.1407308 29172781 10.1080/09513590.2017.1407308

[14] Adami G, Giollo A, Rossini M et al (2020) Different fracture risk profile in patients treated with anti-osteoporotic drugs in real-life. Reumatismo 72:71–74. 10.4081/reumatismo.2020.1267 32700872 10.4081/reumatismo.2020.1267

[15] Porcu G, Biffi A, Ronco R et al (2023) Refracture following vertebral fragility fracture when bone fragility is not recognized: summarizing findings from comparator arms of randomized clinical trials. J Endocrinol Invest 47:795–818. 10.1007/s40618-023-02222-0 37921990 10.1007/s40618-023-02222-0 PMC10965723

[16] Kanis JA, McCloskey EV, Johansson H et al (2013) European guidance for the diagnosis and management of osteoporosis in postmenopausal women. Osteoporos Int 24:23–57. 10.1007/s00198-012-2074-y 23079689 10.1007/s00198-012-2074-y PMC3587294

[17] Barron RL, Oster G, Grauer A et al (2020) Determinants of imminent fracture risk in postmenopausal women with osteoporosis. Osteoporos Int 31:2103–2111. 10.1007/s00198-020-05294-3 32613410 10.1007/s00198-020-05294-3 PMC7560920

[18] Leib ES, Saag KG, Adachi JD et al (2011) Official positions for FRAX® clinical regarding glucocorticoids: the impact of the use of glucocorticoids on the estimate by FRAX® of the 10 year risk of fracture. J Clin Densitom 14:212–219. 10.1016/j.jocd.2011.05.014 21810527 10.1016/j.jocd.2011.05.014

[19] Hadji P, Aapro M, Al-Dagri N et al (2025) Management of aromatase inhibitor-associated bone loss (AIBL) in women with hormone-sensitive breast cancer: an updated joint position statement of the IOF, CABS, ECTS, IEG, ESCEO, IMS, and SIOG. J Bone Oncol 53:100694. 10.1016/j.jbo.2025.100694 40726588 10.1016/j.jbo.2025.100694 PMC12301826

[20] Corrao G, Biffi A, Porcu G et al (2023) Executive summary: Italian guidelines for diagnosis, risk stratification, and care continuity of fragility fractures 2021. Front Endocrinol. 10.3389/fendo.2023.1137671 10.3389/fendo.2023.1137671 PMC10151776 37143730

[21] Wang M, Seibel MJ (2023) Approach to the patient with bone fracture: making the first fracture the last. J Clin Endocrinol Metab 108:3345–3352. 10.1210/clinem/dgad345 37290052 10.1210/clinem/dgad345 PMC10655538

