# Supplementary material for: Under-recognized, under-referred: a multidisciplinary evaluation of fragility fracture management in the emergency setting
Source: Arch Osteoporos. 2026 Jun 18;21(1):93. doi: 10.1007/s11657-026-01684-y (PMC13279481; doi:10.1007/s11657-026-01684-y)
Supplement: Supplementary file 1 — Supplementary file1 (DOCX 16.3 KB) [file 11657_2026_1684_MOESM1_ESM.docx]

**Supplementary Materials 1**

*Survey questions administered to emergency medicine physicians, emergency department nurses, and radiologists*

**1.1.** *Is the mechanism and location of the accident investigated (e.g., fall from standing height, minor trauma, or more severe accident)?*A. Always
B. In women, regardless of age
C. Only in women > 50 years
D. In all individuals (men and women) > 50 years
E. Sometimes
F. Rarely / never
G. Other: please specify _______________

**1.2.** *Is a history of recent or frequent falls, and the mechanism of the fall, investigated?*
A. Always
B. In women, regardless of age
C. Only in women > 50 years
D. In all individuals > 50 years
E. Sometimes
F. Rarely / never
G. Other: please specify _______________

**1.3.** *Is there an investigation into a history of frequent or recent fractures, and how they occurred?*

A. Always
B. In women, regardless of age
C. Only in women > 50 years
D. In all individuals > 50 years
E. Sometimes
F. Rarely / never
G. Other: please specify _______________

**1.4.** *In the case of previous fractures, which characteristic elements are investigated?*A. Number of fractures in medical history
B. Age at the time of the fracture(s)
C. Site of the fracture
D. Cause of the fracture(s)
E. Diagnoses made after the fracture(s)
F. Treatments received after the fracture(s)
G. None of the above

**1.5.** *Do you investigate whether there are cases of hip or other fractures in the family, such as in first-degree relatives?*

A. Always
B. In women, regardless of age
C. Only in women > 50 years
D. In all individuals > 50 years
E. Sometimes
F. Rarely / never
G. Other: please specify _______________

**1.6.** *Is it investigated whether the patient is experiencing or has experienced early menopause?
A. Always*B. Never
C. Sometimes
D. Other: please specify _______________

**1.7.** *During the emergency department visit, is it investigated whether a diagnosis of bone fragility has been made?*
A. Always
B. In women, regardless of age
C. Only in women > 50 years
D. In all individuals > 50 years
E. Sometimes
F. Rarely / never
G. Other: please specify _______________

**1.8.** *Is it investigated whether the patient has ever undergone a specialist evaluation for osteoporosis?*
A. Always
B. Sometimes
C. Never
D. Other: please specify _______________

**1.9.** *Is it investigated whether the patient has ever received a diagnosis of osteoporosis or has undergone treatments for bone health?*
A. Always
B. In women, regardless of age
C. Only in women > 50 years
D. In all individuals > 50 years
E. Never
F. Other: please specify _______________

**1.10.** *Which medications or treatments for osteoporosis has the patient taken in the past or is currently taking, and with what level of adherence? Is this question asked:*
A. Always
B. In women, regardless of age
C. Only in women > 50 years
D. In all individuals > 50 years
E. Never
F. Other: please specify _______________

**1.11.** *Has the patient taken or is currently taking vitamin D, and with what level of adherence? Is this question asked:*
A. Always
B. In women, regardless of age
C. Only in women > 50 years
D. In all individuals > 50 years
E. Never
F. Other: please specify _______________

**1.12.** *Is it investigated whether the patient has ever undergone bone densitometry (DEXA)?
A. Always*B. In women, regardless of age
C. Only in women > 50 years
D. In all individuals > 50 years
E. Rarely / never
F. Sometimes
G. Other: please specify _______________

**1.13.** *Is the patient’s medication history investigated as a risk factor for fractures (with particular attention to steroids, anticonvulsants, or adjuvant anti-hormonal therapies for cancer)?*
A. Always
B. In women, regardless of age
C. Only in women > 50 years
D. In all individuals > 50 years
E. Rarely / never
F. Other: please specify _______________

**1.14.** *Is it investigated whether there is frequent alcohol or tobacco use?*
A. Always
B. In women, regardless of age
C. Only in women > 50 years
D. In all individuals > 50 years
E. Rarely / never
F. Sometimes
G. Other: please specify _______________

**1.15*.*** *Does the patient present difficulty walking or a reduced gait speed?*
A. Always
B. In women, regardless of age
C. Only in women > 50 years
D. In all individuals > 50 years
E. Rarely / never
F. Other: please specify _______________

**1.16.** *Do you investigate whether the patient has ever been afraid of falling or has noticed difficulty maintaining balance?*

A. Always
B. In women, regardless of age
C. Only in women > 50 years
D. In all individuals > 50 years
E. Rarely / never
F. Other: please specify _______________

**1.17.** *Is it investigated whether there are tripping hazards in the home (e.g., clutter, obstacles, pets, rugs)?*A. Always
B. In women, regardless of age
C. Only in women > 50 years
D. In all individuals > 50 years
E. Rarely / never
F. Other: please specify _______________

**1.18.** (Radiologist) *How often do you report osteoporosis in relation to the fracture you observe (i.e., associate the type of fracture with bone fragility)?*
A. Always
B. Sometimes
C. Rarely / never
D. Other: please specify _______________

**1.19.** (Radiologist) *How often is the degree of vertebral fracture quantified?*
A. Always
B. Sometimes
C. Rarely / never
D. Other: please specify _______________

**1.20.** (Emergency Medicine Physician) *How often are certain types of fractures (e.g., distal radius, proximal humerus, femur) indicated or recognized as strongly suspected osteoporotic fractures?*
A. Always
B. Sometimes
C. Rarely / never
D. Other: please specify _______________

**1.21.** (Emergency Medicine Physician) How often is “fragility fracture” indicated at the time of discharge?
A. Always
B. Sometimes
C. Never
D. Other: please specify _______________
